# Astragaloside IV Attenuated 3,4-Benzopyrene-Induced Abdominal Aortic Aneurysm by Ameliorating Macrophage-Mediated Inflammation

**DOI:** 10.3389/fphar.2018.00496

**Published:** 2018-05-22

**Authors:** Jiaoni Wang, Yingying Zhou, Shaoze Wu, Kaiyu Huang, Saroj Thapa, Luyuan Tao, Jie Wang, Yigen Shen, Jinsheng Wang, Yangjing Xue, Kangting Ji

**Affiliations:** ^1^Department of Cardiology, The Second Affiliated Hospital and Yuying Children’s Hospital of Wenzhou Medical University, Wenzhou, China; ^2^Department of Endocrinology, The Second Affiliated Hospital and Yuying Children’s Hospital of Wenzhou Medical University, Wenzhou, China

**Keywords:** abdominal aortic aneurysm, 3, 4-benzopyrene, astragaloside IV, macrophage, inflammation, oxidative stress

## Abstract

Abdominal aortic aneurysm (AAA), characterized by macrophage infiltration-mediated inflammation and oxidative stress, is a potentially fatal disease. Astragaloside IV (AS-IV) has been acknowledged to exhibit antioxidant and anti-inflammatory properties. This study was designed to investigate the protective effect of AS-IV against AAA formation induced by 3,4-benzopyrene (Bap) and angiotensin II (Ang II), and to explore probable mechanisms. Results showed that AS-IV decreased AAA formation, and reduced macrophage infiltration and expression of matrix metalloproteinase. Furthermore, AS-IV abrogated Bap-/Ang II-induced NF-κB activation and oxidative stress. *In vitro*, AS-IV inhibition of macrophage activation and NF-κB was correlated with increased phosphorylation of phosphatidylinositol 3-kinase (PI3-K)/AKT. Together, our findings suggest that AS-IV has potential as an intervention in the formation of AAA.

**Highlights:**

(1) The protective effect of Astragaloside IV (AS-IV) on abdominal aortic aneurysm (AAA) is associated with its suppressing effects on inflammation in the aortic wall.
(2) AS-IV abrogated 3,4-benzopyrene (Bap)/angiotensin II (Ang II)-induced nuclear factor-κB (NF-κB) activation and oxidative stress.
(3) AS-IV inhibited Bap-induced RAW264.7 macrophage cells activation by inhibiting oxidative stress and NF-κB activation through phosphatidylinositol 3-kinase (PI3-K)/AKT pathway.
AS-IV is a potential preventive agent for cigarette smoking-related AAA.

## Introduction

Abdominal aortic aneurysm (AAA) is a life-threatening vascular disease, predominantly associated with risk factors that include age, male gender, and smoking. It results in mortality in about 90% of cases (Golledge et al., 2006; Hellenthal et al., 2009a,b). It is defined by local expansion of the abdominal aorta greater than 50% of the normal diameter (Pennell et al., 1985), with chronic inflammation and degradation of elastin and collagen as its main features (Cao et al., 2010). In spite of numerous advances in AAA imaging and surgery, most patients do not receive treatment, due to the low benefit-to-risk ratio of surgical interventions, especially among patients with small (5.5 cm for males, 5 cm for females), asymptomatic AAA (Brewster et al., 2003; Baxter et al., 2008). Hence, non-invasive therapies are urgently needed.

It has been widely acknowledged that inflammation and oxidative stress can induce AAA (Shimizu et al., 2006; McCormick et al., 2007). AAA can be roughly divided into two pathophysiological processes, inflammation and extracellular matrix degeneration (Freestone et al., 1995), both of which are associated with macrophages. Macrophages secrete monocyte chemoattractant protein-1 to recruit monocytes and additional macrophages to the vessel wall, and produce proinflammatory cytokines and proteases that maintain chronic vascular inflammation and extracellular matrix destruction, leading to AAA formation (Daugherty and Cassis, 2004; Shimizu et al., 2006; Lai C.H. et al., 2013; Wang et al., 2013). Numerous animal studies have demonstrated that this involves significantly elevated levels of reactive oxygen species (ROS) in the aortic wall (McCormick et al., 2007), which activate matrix metalloproteinases (MMPs) and destroy elastin lamellae (Rajagopalan et al., 1996). Inflammatory responses can promote the development of aneurysms by enhancing the production of ROS (Miller et al., 2002); therefore, reducing ROS production and inflammation may attenuate AAA (Cao et al., 2010; Wu et al., 2011).

3,4-Benzopyrene (Bap) is a polycyclic aromatic hydrocarbon composed of five benzene rings and a representative component of fine particles (PM2.5) in atmospheric pollutants and tobacco smoke (Zhang et al., 2017), widely existing in all kinds of chemical industry exhaust gas and domestic sewage. Epidemiological investigations have shown that exposure to smoke is associated with the development of AAA and smoking is one of the risk factors for AAA (Erbel et al., 2014; LeFevre and U.S. Preventive Services Task Force, 2014). Recent studies have found that Bap may have more potential targets and can affect the expression and activity of key proteins in multiple signaling pathways (Verma et al., 2012). Animal experiments confirmed the role of Bap on the basis of the classical angiotensin II (Ang II)-induced AAA mouse model (Daugherty and Cassis, 2004). Our work and others confirmed that compared with the pure Ang II group, Bap/Ang II can significantly increase the incidence of AAA (Zhang and Ramos, 2008; Ji et al., 2014).

Astragaloside IV (AS-IV) is a Chinese herbal medicine extracted from the root of *Astragalus membranaceus Bunge*, which has been reported to be effective against a variety of diseases in preclinical studies (Luo et al., 2004; Yu et al., 2006; Zhang et al., 2006; Du et al., 2008; Liu et al., 2009; Guo et al., 2016). In China, AS-IV has long been used for therapeutic purposes in cardiovascular disease (Du et al., 2008), including the treatment of arrhythmias and the improvement of ventricular function in patients suffering from ischemic heart disease (Zhang et al., 2006). The cardioprotective action of AS-IV is mainly due to its anti-inflammatory and antioxidant effects (Lai P.K. et al., 2013; Zhao et al., 2013). Although oxidative stress and inflammation are known to be involved in the development of AAA, no previous research has determined whether AS-IV might have a protective effect.

In this study, we tested the hypothesis that AS-IV protects against Bap-induced AAA by inhibiting inflammation and reducing oxidative stress. Further, we explored likely mechanisms, and found that the protective effect of AS-IV in AAA may correlate with upregulated phosphatidylinositol 3-kinase (PI3-K)/AKT phosphorylation. The results support our hypothesis and suggest that AS-IV may be a safe and effective drug candidate for AAA.

## Materials and Methods

### Materials

Bap and AS-IV were of high purity (98%) and were purchased from Sigma-Aldrich (St. Louis, MO, United States) and the National Institute for the Control of Pharmaceutical and Biological Products (Beijing, China), respectively. For cell culture experiments, they were dissolved in dimethyl sulfoxide (DMSO) at a stock concentration of 50 mg/ml and diluted to final concentration in culture medium.

### Animal and Experimental Protocol

The Animal Care and Use Committee at the Wenzhou Medical College approved the study (NO: wzdw2015-0007). Animal care complied with the Guide for the Care and Use of Laboratory Animals. Male C57/B6j mice, 8–10 months old, weighing 35–40 g (Weitong Lihua Experimental Animal Technology Co. Ltd., Beijing, China), were housed in a specific pathogen-free environment, with normal mouse chow and water provided *ad libitum*. Mice were divided into four groups. The control group received a weekly intraperitoneal injection of medium-chain triglycerides. Mice in the Bap/Ang II group received Ang II infusion (0.90 mg/kg/day, Sigma-Aldrich) via a subcutaneous osmotic minipump (Alzet Osmotic Pump, Model 2006; DURECT Corporation, Cupertino, CA, United States) and a weekly intraperitoneal injection of Bap (10 mg/kg, dissolved in 2 mg/ml medium-chain triglycerides). The AS-IV group received 20 or 80 mg/kg AS-IV intragastrically once daily, in addition to the above Ang II and Bap treatments. Osmotic minipumps were inserted subcutaneously, as previously described (Ji et al., 2014). After 6 weeks, mice were euthanized.

### AAA Evaluation

After euthanasia, the abdominal and thoracic cavities of each of the mice were exposed. Phosphate-buffered saline (PBS), followed by 4% formaldehyde, was perfused through the left ventricle into the aorta. Abdominal aortic tissue sections were removed, between the ileal bifurcation and the last intercostal artery, and fixed in 4% paraformaldehyde for immunohistochemistry. For each animal, at least 12 aortic sections were selected, diameters were determined, and the average diameter was calculated. AAA was defined as an increase in the average diameter of the aorta by ≥50%.

### Elastin–Van Gieson, Immunofluorescence, and Immunohistochemistry

Macroscopic examination of removed tissues was performed, and suprarenal abdominal aortas were used for histological analysis. Tissue segments were submerged in 4% paraformaldehyde for fixation, embedded in paraffin, and sectioned (5 μm). Sections of abdominal aorta (5 μm thick) were deparaffinized and rehydrated, then stained with elastin–Van Gieson (EVG) to assess the integrity of elastin layers. To detect target protein expression, primary antibodies against CD68 (1:500; ab53444, Abcam, Shanghai, China), NF-κBp65 (1:500; ab32536, Abcam, Shanghai, China), MMP-12 (1:500; ab66157, Abcam, Shanghai, China), and tumor necrosis factor (TNF)-α (1:200; ab6671, Abcam, Shanghai, China) were used. Nuclei were stained with 4′,6-diamidino-2-phenylindole or 3′,3-diaminobenzidine. Identical camera settings were used for all images.

### Measurement of Reactive Oxygen Species in AAA

Following euthanasia, aortas were perfused with cold PBS through the left ventricle. Abdominal aortas were removed, buried in optimum cutting temperature compound, and immediately frozen. Freshly prepared frozen sections were soaked in PBS for 30 min and incubated with the fluorescent dye dihydroethidium (DHE; 5 μM) in a dark, humidified chamber at 37°C for 30 min.

### Cell Culture and AS-IV Treatment

The mouse macrophage cell line RAW264.7 cells were obtained from ATCC. Cells were grown in culture bottles and maintained in Dulbecco’s modified Eagle’s medium (DMEM) supplemented with 10% fetal bovine serum. Cultures were divided into six treatment groups: (1) control, incubated in DMEM; (2) solvent control (DMSO), incubated in DMEM containing 0.1% DMSO; (3) Bap stimulation (Bap), incubated in DMEM containing 20 μM Bap for 24 h; (4–6) low-(AL), medium-(AM), and high-dose (AH) AS-IV, pretreated for 2 h with 2, 10, and 50 μg/ml AS-IV, respectively, followed by co-incubation with 20 μM Bap for 24 h.

### Immunocytochemistry

Immunocytochemistry staining was performed to assess nuclear translocation of NF-κB. RAW264.7 cells were pretreated with or without AS-IV (10 μg/ml) for 2 h prior to exposure to Bap (20 μM) for 24 h. Cells were washed with PBS and fixed in 4% paraformaldehyde for 15 min. After loading the vector, the cells were treated with 0.1% Triton X-100 and blocked with 5% bovine serum albumin. After 30 min, they were incubated overnight with NF-κBp65 (1:250; ab32536, Abcam, Shanghai, China) antibody at 4°C, followed by a fluorescein-conjugated secondary antibody.

### Measurement of Reactive Oxygen Species in RAW264.7 Cells

Dichlorodihydrofluorescein diacetate (DCFH-DA), a fluorescent probe, was used to measure the intracellular accumulation of ROS. DCFH-DA is converted into dichlorodihydrofluorescein (DCFH) by intracellular deacetylases, and then oxidized to dichlorofluorescein (DCF) by various ROS within cells. DCF is a highly fluorescent compound. Cells were subjected to different treatments, washed with PBS, and stained with 10 μM DCFH-DA for 20 min in the dark at room temperature. Cells were then harvested and intracellular ROS production was analyzed using flow cytometry.

### Statistical Analysis

Data are expressed as the mean ± standard deviation. All outcomes are compared among groups using one-way ANOVA followed by Dunnett’s multiple comparison test. Statistical analyses were performed using SPSS 14 software (SPSS, Inc.). Values of *P* < 0.05 were considered statistically significant.

## Results

### AS-IV Treatment Inhibits Bap-/Ang II-Induced AAA in C57/B6j Mice

**FIGURE 1:**
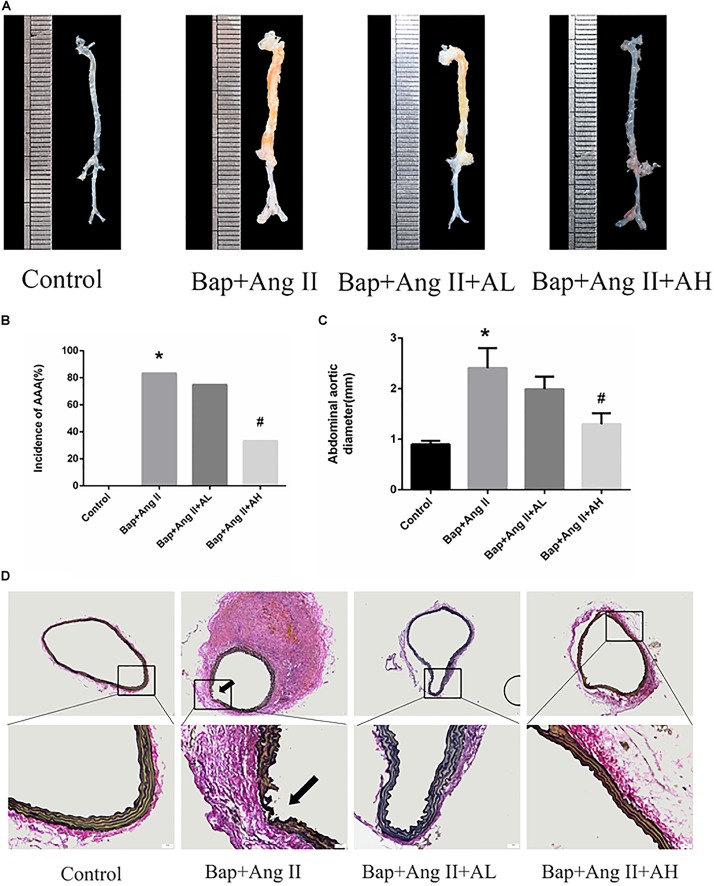
Astragaloside IV (AS-IV) treatment inhibits 3,4-benzopyrene (Bap)/angiotensin II (Ang II)-induced abdominal aortic aneurysm (AAA) in C57/B6j mice. Mice were treated with vehicle (*n* = 5), Bap + Ang II (*n* = 12), Bap + Ang II + 20 mg/kg AS-IV (*n* = 12), and Bap + Ang II + 80 mg/kg AS-IV (*n* = 12). **(A)** Representative images of abdominal aortic specimens 42 d after induction. **(B)** Incidence of AAA in the control, Bap/Ang II, and two AS-IV treatment groups. **(C)** Maximal external diameter of suprarenal aorta (mm) was measured by morphometry at day 42. Data represent the mean ± SD. ^∗^*P* < 0.05 vs. control group; ^#^*P* < 0.05 vs. Bap + Ang II group. **(D)** Representative photo of elastin–Van Gieson (EVG) staining in the control, Bap/Ang II, and two doses of AS-IV treatment groups. Arrows indicate disrupted elastin fibers. Scale bar, 20 μm.

We first investigated whether AS-IV had an effect on Bap-/Ang II-induced AAA in C57/B6j mice. Representative photographs are shown in **Figure 1A**. Macroscopic observation revealed that the aortic diameter was increased significantly in the Bap/Ang II group, which was significantly reduced in mice treated with high doses of AS-IV. The incidence of AAA was 83.3, 75, and 33.3% in the Bap-/Ang II-treated mice receiving no AS-IV, low-dose AS-IV, and high-dose AS-IV, respectively, while AAA was not observed in the control group (**Figure 1B**). The incidence of AAA was significantly reduced in the high-dose AS-IV group (**Figures 1A,B**), whereas low-dose AS-IV had no significant effect. Similarly, abdominal aortic diameters of Bap-/Ang II-treated mice were greatly reduced in the high-dose AS-IV group (**Figure 1C**) but not in the low-dose group. EVG staining revealed that Bap, combined with Ang II infusion, led to aortic media and adventitia breakdown, adventitia hypertrophy, and elastin fiber destruction and discontinuity. These pathological changes were largely decreased in the high-dose AS-IV group (**Figure 1D**). Aortic morphology was similar in the Bap/Ang II and low-dose AS-IV groups.

### AS-IV Inhibits Macrophage Infiltration in the Aortic Wall of Bap-/Ang II-Treated Mice

**FIGURE 2:**
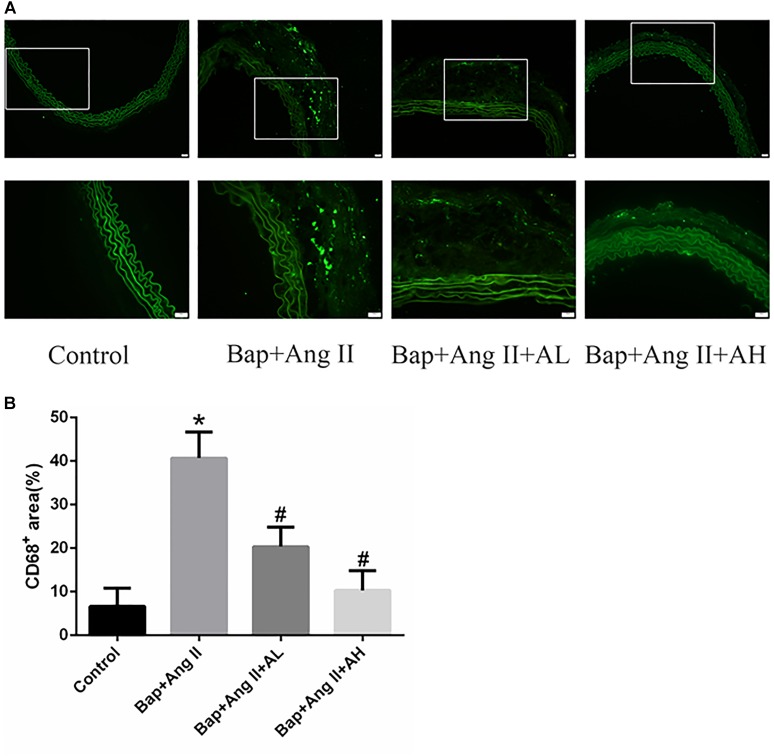
AS-IV inhibits macrophage infiltration in the aortic wall of Bap-/Ang II-treated mice. Mice were subjected to different treatments and AAAs were harvested for histological analysis. **(A)** Representative photomicrographs of CD68+ cells staining in suprarenal aortic sections: immunoreactivity was visualized using an Alexa Fluor 488 secondary antibody (green). **(B)** CD68^+^ area (%) in suprarenal aortic sections. *n* = 6 for each group. ^∗^*P* < 0.05 vs. control group; ^#^*P* < 0.05 vs. Bap + Ang II group. Scale bar, 20 μm.

Given that macrophages are the major inflammatory cells in AAA, and that their infiltration into the aneurysmal aortic wall is characteristic of AAA pathology, we used immunofluorescence staining to examine the expression of CD68 in aortic tissues. This revealed that macrophage infiltration (**Figures 2A,B**) was significantly decreased by high-dose AS-IV from that seen in the Bap-/Ang II-treated group. We therefore focused on macrophages to elucidate the possible mechanisms of AS-IV action against Bap-/Ang II-induced AAA.

### AS-IV Inhibits NF-κB Activation and Inflammation in the Aortic Wall of Bap-/Ang II-Treated Mice

**FIGURE 3:**
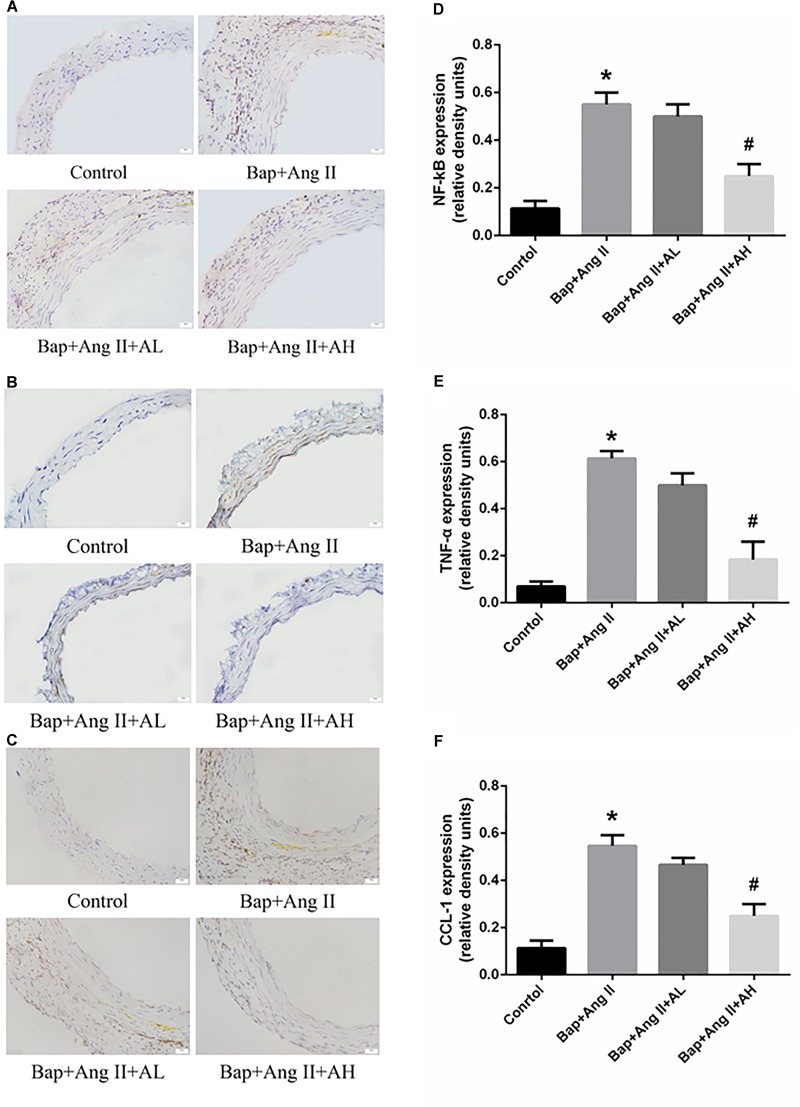
AS-IV inhibits nuclear factor-κB (NF-κB) activation and proinflammatory cytokines and chemokines production in Bap-/Ang II-treated mice. **(A–C)** Representative immunohistochemistry analysis of NF-κB, TNF-α, and C–C motif chemokine (CCL)-1 expression in abdominal aortas of the control, Bap/Ang II, and two AS-IV treatment groups. Scale bar, 20 μm. **(D–F)** Quantitative analysis of NF-κB, TNF-α, and CCL1 expression in four groups of mice. *n* = 5 for each group. ^∗^*P* < 0.05 vs. control group; ^#^*P* < 0.05 vs. Bap + Ang II group.

Chronic inflammation of the aortic wall is a primary feature of AAA. As expected, we observed upregulated expression of NF-κB and proinflammatory cytokines, such as TNF-α and chemokine (C–C motif) ligand-1 (CCL-1), in Bap-/Ang II-treated C57/B6j mice. As shown by immunostaining, protein expression of NF-κB, CCL-1, and TNF-α was remarkably attenuated by high-dose AS-IV but not by low-dose AS-IV. Representative photomicrographs of aortas from these animals are shown in **Figures 3A–C**; **Figures 3D–F** show the quantitative analyses of these results.

### AS-IV Treatment Reduces ROS Levels and MMP Expression in the Aortic Wall of Bap-/Ang II-Treated Mice

**FIGURE 4:**
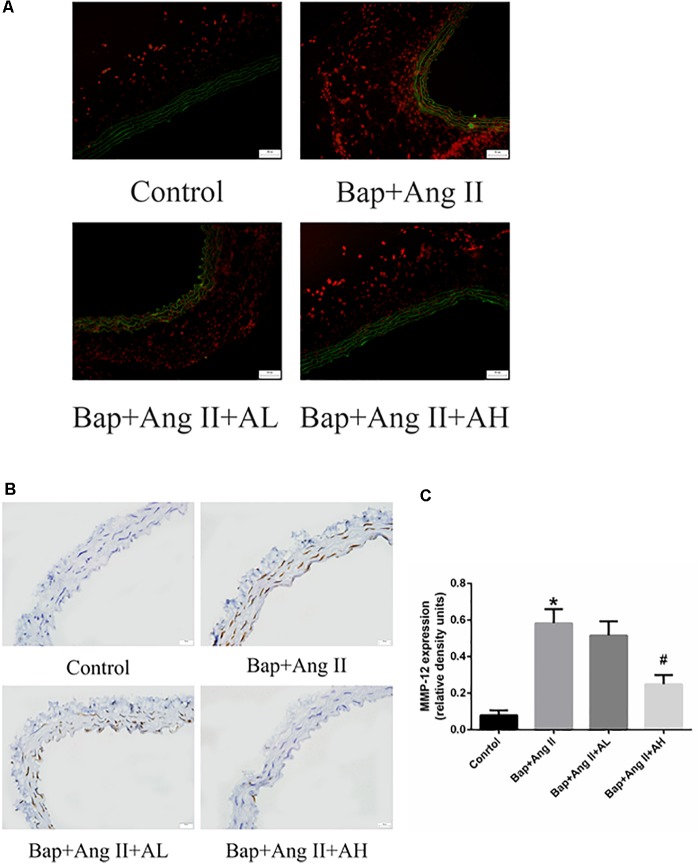
AS-IV treatment reduces reactive oxygen species (ROS) levels and matrix metalloproteinase (MMP) expression in the aortic wall of Bap-/Ang II-treated mice. The mice were subjected to different treatments to obtain the aorta. Dihydroethidium (DHE) staining (red fluorescence) was performed to assess ROS levels. **(A)** Representative images of DHE staining from abdominal aneurysmal segment of control, Bap/Ang II, and two AS-IV treatment groups. **(B)** Representative images of immunohistochemistry for MMP-12 in AAA of four groups of mice. Scale bar, 20 μm. **(C)** Quantitative analysis of MMP-12 in four groups of mice. *n* = 5/group. ^∗^*P* < 0.05 vs. control group; ^#^*P* < 0.05 vs. Bap + Ang II group.

Numerous studies have implicated a critical role for ROS in AAA formation. To assess ROS levels, DHE staining was used. Intense staining was observed in the aortic walls of Bap-/Ang II-treated mice, indicating robust production of ROS. High-dose AS-IV was associated with significantly decreased ROS levels, whereas low-dose AS-IV was not (**Figure 4A**). We further analyzed the expression of MMP-12 in aortic tissues to evaluate the effect of AS-IV on extracellular matrix homeostasis. Compared with the Bap/Ang II group, mice receiving high-dose AS-IV showed profoundly reduced MMP-12 protein levels (**Figures 4B,C**).

### AS-IV Reduces ROS as Well as MMP Protein and Proinflammatory Cytokines Expression in Bap-Induced RAW264.7 Cells

**FIGURE 5:**
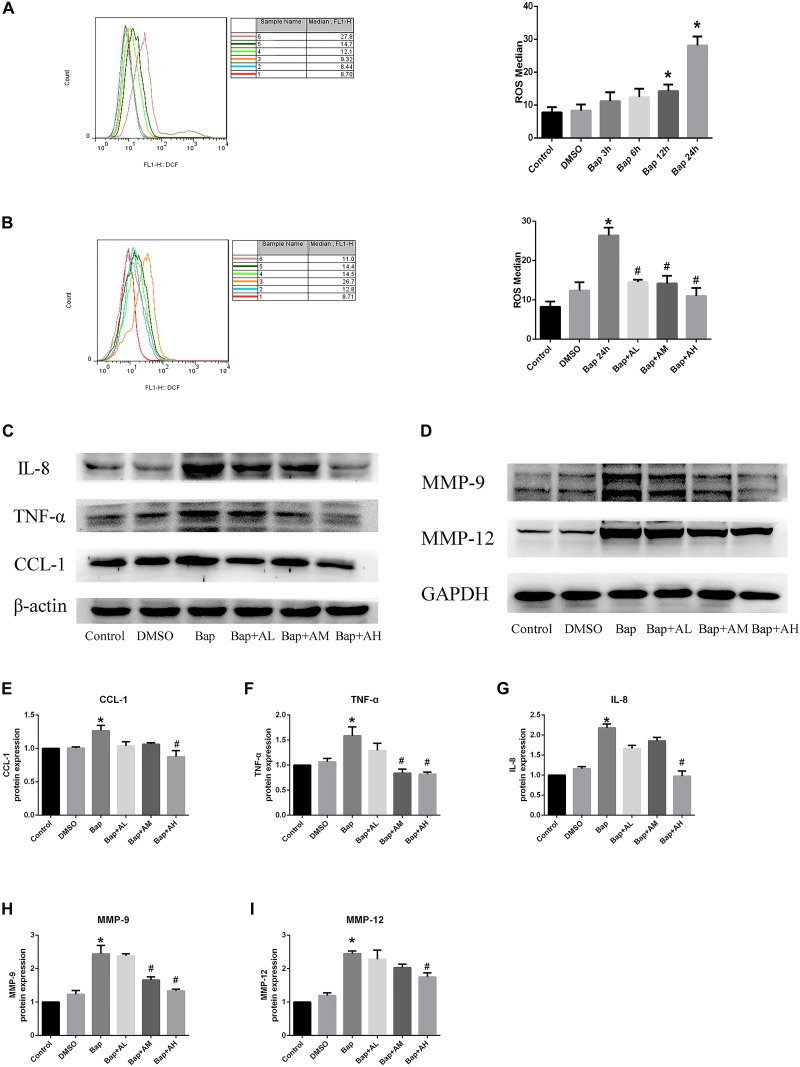
AS-IV reduces reactive oxygen species (ROS), MMP protein, and proinflammatory cytokines expression in Bap-induced Raw264.7 cells. **(A)** Typical images of ROS expression in raw264.7 cells after exposed to 20 μM Bap for 3, 6, 12, and 24 h, respectively. **(B)** Typical images of ROS expression in raw264.7 cells. The cells were preincubated with 2, 10, or 50 μg/ml AS-IV for 2 h, thereafter incubated with 20 μM Bap for 24 h. The intracellular accumulation of ROS was measured by FACS flow cytometry. Data are representative of three independent experiments with determinations. ^∗^*P* < 0.05 compared with control; ^#^*P* < 0.05 vs. Bap group. **(C)** Representative Western blot analysis of C–C motif chemokine (CCL)-1, interleukin (IL)-8, and TNF-α in control, DMSO, Bap, and three doses of AS-IV treatment cells. **(D)** Representative Western blot analysis of MMP-9 and MMP-12 in six groups. **(E–I)** Quantitative analysis of CCL1, IL-8, TNF-α, MMP-9, and MMP-12 expression in six groups of cells. *n* = 6/group. ^∗^*P* < 0.05 compared with control; ^#^*P* < 0.05 vs. Bap group.

After exposure of RAW264.7 cells to Bap without AS-IV pretreatment, flow cytometry indicated that intracellular accumulation of ROS was significantly increased. Bap-induced ROS production was time-dependent, becoming evident after 12 h (**Figure 5A**). As expected, Bap-induced ROS production was significantly suppressed by AS-IV pretreatment in a dose-dependent manner (**Figure 5B**). Moreover, high-dose AS-IV dramatically blunted Bap-induced upregulation of MMPs in RAW264.7 cells (**Figure 5D**). In addition, Bap caused a twofold increase in the protein levels of CCL-1, interleukin-8, and TNF-α at 24 h, compared with control cultures (**Figure 5C**). However, AS-IV significantly decreased the expression of these proinflammatory cytokines in cells after Bap exposure. These results show that AS-IV significantly inhibited the Bap-induced overexpression of inflammation-associated proteins in RAW264.7 cells. **Figures 5E–I** show the quantitative analysis of these results.

### AS-IV Inhibits NF-κB Activation in RAW264.7 Cells Following Bap Exposure

**FIGURE 6:**
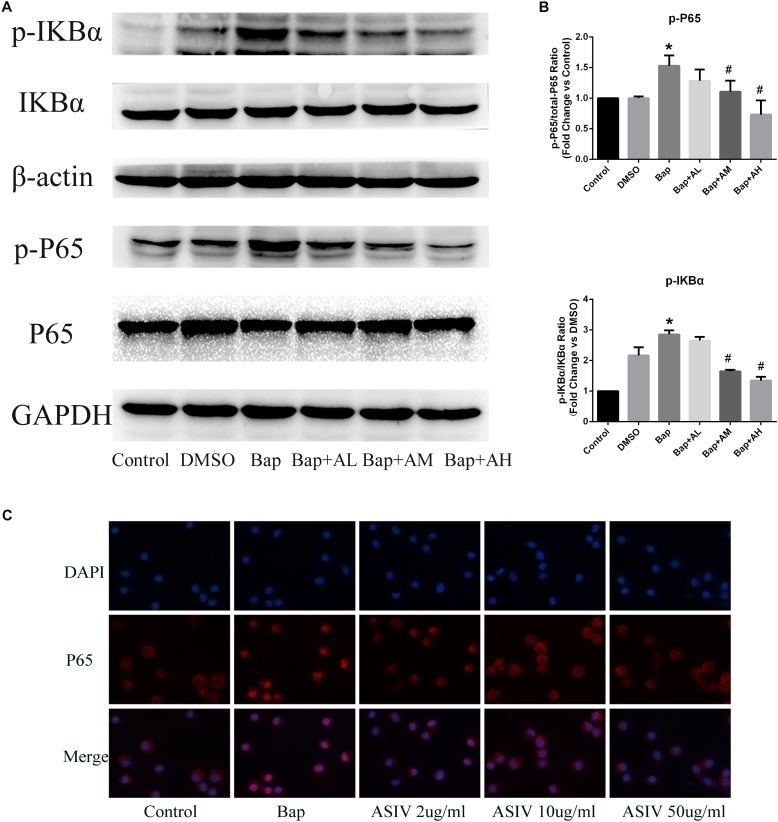
AS-IV inhibits Bap-induced NF-κB activation. After exposure of the RAW 264.7 cells to Bap with or without AS-IV pretreatment, the cells were collected. **(A)** Representative Western blot analysis of phospho(p)-P65 and p-IκBα. **(B)** p-P65/P65 and p-IκBα/IκBα ratios were expressed as the relative value of the control. *n* = 6/group. ^∗^*P* < 0.05 vs. control group; ^#^*P* < 0.05 vs. Bap group. **(C)** The localization of P65 was visualized by immunofluorescence analysis as described under the section “Materials and Methods.” *n* = 3/group. magnification: ×400.

We investigated whether Bap-induced translocation of NF-κB into the nucleus could be suppressed by AS-IV. As shown in **Figure 6C**, NF-κB was detectable only in the cytoplasm of untreated cells. When macrophages were exposed to Bap (20 μM) for 24 h, translocation from the cytoplasm into the nucleus occurred. However, this translocation was significantly inhibited by 50 μg/ml AS-IV. Furthermore, western blotting revealed that the expression level of phosphorylated P65 (p-P65) and p-IκB was significantly suppressed by high-dose AS-IV treatment (**Figure 6A**). **Figure 6B** shows the quantitative analysis of these results.

### Effect of AKT Inhibitor LY294002 on the Expression of p-AKT and p-P65 in Bap-Treated RAW264.7 Cells

**FIGURE 7:**
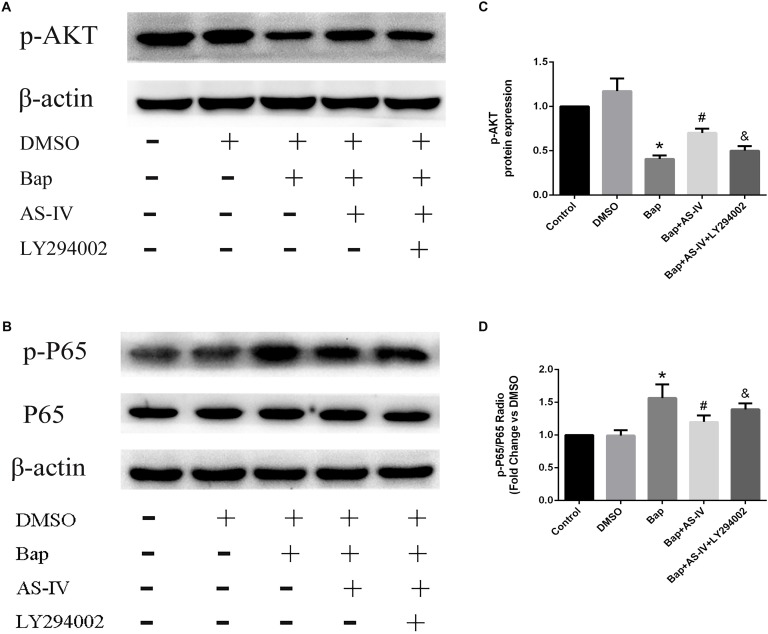
Effect of AKT inhibitor LY294002 on the expression of phospho(p)-AKT, p-P65 in Bap-stimulated RAW264.7 cells. **(A,B)** Representative Western blot analysis of p-AKT, p-P65 in five groups of cells. **(C,D)** Quantitative analysis of p-AKT and p-P65 expression normalized to β-actin level. *n* = 6/group. ^∗^*P* < 0.05 vs. control group; ^#^*P* < 0.05 vs. Bap group; ^&^*P* < 0.05 vs. Bap + AS-IV group.

To confirm the signaling proteins participating in the AS-IV effect, we studied the *in vitro* expression level of AKT and NF-κB. Notably, RAW264.7 cells treated with high-dose AS-IV were found to have significantly elevated p-AKT production and reduced p-65 expression when compared with Bap-treated cultures (**Figures 7A,B**). To further study the role of AKT in the effect of AS-IV, we pretreated RAW264.7 cells with the AKT inhibitor LY294002. Blunting AKT signaling eliminated the suppression of inflammation by AS-IV (**Figures 7A,B**). Therefore, AKT may act as a key regulator in the protective effect of AS-IV on Bap-treated RAW264.7 cells. **Figures 7C,D** show the quantitative analysis of these results.

## Discussion

The present study demonstrates that AS-IV has a protective effect against AAA induced by Bap/Ang II. Decreased AAA formation in Bap-/Ang II-treated animals occurred concomitantly with a reduction of both macrophage infiltration and expression of MMP-12. Furthermore, AS-IV could abrogate Bap-/Ang II-induced oxidative stress and NF-κB activation. We speculate that AS-IV has potential as a preventive agent for cigarette smoking-related AAA.

Smoking has long been considered an important risk factor for AAA (Alcorn et al., 1996). Cigarettes contain compounds that contribute to AAA formation, and one of the key components is Bap (Guo et al., 2010). Our group previously demonstrated that Bap could work synergistically with Ang II in a mouse AAA model (Ji et al., 2014). We suggest that Bap promotes AAA formation by promoting infiltration of macrophages, activating NF-κB, and upregulating the expression of MMP-9 and MMP-12.

Inflammation and oxidative stress are vital factors in AAA development (Shimizu et al., 2006; McCormick et al., 2007). Chronic inflammation, featuring inflammatory cell infiltration and proinflammatory cytokine expression, was observed in Bap-/Ang II-induced AAA (Shimizu et al., 2006; Zhang et al., 2009). Inflammatory cells, especially macrophages, are involved in the initiation of AAA by tobacco smoke (Daugherty and Cassis, 2004). Therefore, targeting macrophage-mediated vascular inflammation may be a potential treatment for the prevention of AAA (Liu et al., 2012; Trivedi et al., 2013). Macrophages represent circulating members of the myeloid cell lineage (Ginhoux and Jung, 2014), and participate in inflammation and matrix degradation by releasing proinflammatory cytokines and matrix-degrading proteases (Kaneko et al., 2011; Wang et al., 2015). High-dose AS-IV treatment significantly attenuated macrophage accumulation in AAA tissues and markedly decreased the expression of proinflammatory cytokines and chemokines. In RAW264.7 cells, we demonstrated that AS-IV could dose-dependently inhibit NF-κB-mediated inflammatory signaling pathways. These outcomes demonstrate that the protective role of AS-IV against AAA is related to its suppressive effects on inflammation in the aortic wall.

The level of ROS is significantly higher in AAA segments than in adjacent non-aneurysmal aorta, in both humans and experimental animals, suggesting that ROS is a key participant in AAA (Ho et al., 2016). Concomitant with AAA, ROS levels were markedly increased in the aortic wall of Bap/Ang II mice. As expected, AS-IV significantly inhibited AAA formation, and the ROS-scavenging function of AS-IV was confirmed in Bap-treated RAW264.7 cells. Macrophages on the aortic wall of aneurysms have been identified as the primary source of oxidative stress (McCormick et al., 2007; Tazume et al., 2012; Daugherty and Powell, 2014; Li et al., 2014). Our study confirms this view, both *in vitro* and *in vivo*. ROS can induce macrophages to release proinflammatory cytokines, and, in turn, these cytokines can activate macrophages to increase intracellular ROS (Forman and Torres, 2002; McNelis and Olefsky, 2014). The effect of AS-IV on AAA development may be achieved by reducing the production of proinflammatory cytokines and ROS.

Nuclear factor-κB is an important transcription factor that orchestrates the production of proinflammatory mediators in activated macrophages, and is closely associated with chronic inflammatory disease (Calzado et al., 2007; Lawrence, 2009). NF-κB dimers bind to IκB proteins to form an inactive state. When the IκB kinase complex is activated, it causes the degradation of IκB. Subsequently, the released dimers will transfer from the cytoplasm to the nucleus, and initiate transcription of target genes (Liu et al., 2016). We found that Bap promoted the secretion of downstream factors by enhancing the phosphorylation of IκB and NF-κB in RAW264.7 cells, and that these effects could be eliminated by AS-IV treatment. *In vivo*, we found that the expression of NF-κB in Bap-/Ang II-treated mice was significantly attenuated by high-dose AS-IV treatment.

Excessive extracellular matrix degradation and aortic wall remodeling are the chief pathological characteristics of AAA (Gong et al., 2008). MMPs frequently induce damage of the medial elastic lamellae as a preliminary event in AAA. In particular, the expression and activation of MMP-9 and MMP-12 are enhanced in human AAA, and are essential to its formation. High-dose AS-IV significantly elevated collagen deposition by inhibiting MMP-12 expression, thus preserving vascular integrity. In RAW264.7 cells, AS-IV dramatically blunted Bap-induced upregulation of MMPs.

**FIGURE 8:**
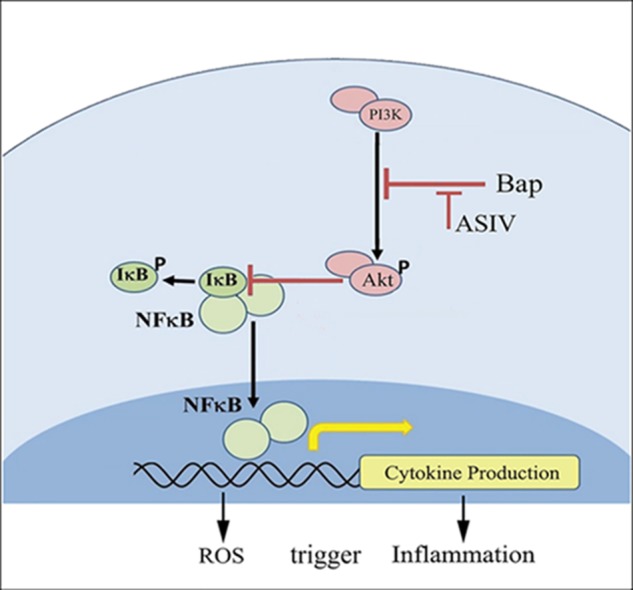
Possible mechanisms by which AS-IV inhibits Bap-induced macrophages activation and inflammatory responses. Bap promotes the secretion of downstream factors and the production of ROS by enhancing the phosphorylation of I-κB and NF-κB. Among them, ROS can induce macrophages to release proinflammatory cytokines, and, in turn, these cytokines can activate macrophages to increase intracellular ROS. AS-IV increases phosphorylation of AKT and inhibits NF-κB expression, thereby exerting anti-inflammatory effects.

PI3-K/AKT signaling has multifunctional effects on cellular metabolism, survival, proliferation, and migration (Manning and Cantley, 2007), and it is well established that inflammation can impair AKT signaling (Shen et al., 2006). The function of AKT in inflammation is controversial; although AKT can promote inflammation (Calamito et al., 2010), it has also been demonstrated in many studies to inhibit inflammation (Schabbauer et al., 2004; Yin et al., 2007; Zhang et al., 2007). The expression and activation of AKT are inhibited in human aortic aneurysmal tissues, and knockout of Akt-2 induced deterioration in Ang II-induced AAA (Shen et al., 2013). The interaction between NF-κB and PI3-K/AKT pathways has been reported (Schabbauer et al., 2004). The current study shows that, at high doses, AS-IV can increase phosphorylation of AKT, suppress NF-κB expression, reduce tissue inflammation, and protect against Bap-/Ang II-induced AAA (**Figure 8**). Furthermore, the *in vitro* experiments illustrate that blocking AKT signaling eliminates the beneficial effects of AS-IV, implying that AKT signaling is an essential step in the pathway mediating these effects.

There are some limitations in our study. First, RAW264.7 cells used in our study are a mouse macrophage cell line, exhibiting the characteristics of a number of immortalized cells. Within tissue, murine peritoneal macrophages or murine bone marrow-derived macrophages can respond to activation stimuli and dramatically change their physiology. So, using these cells may better reflect the characteristics of macrophages. Second, crosstalk between macrophage cells and vascular cells is of importance for aneurysm formation. Next, we can study the effect of AS-IV on vascular cells to further clarify the mechanism of action.

## Conclusion

This study demonstrates that AS-IV treatment significantly reduces AAA formation in Bap-/Ang II-treated mice. AS-IV may attenuate Bap-/Ang II-induced vascular wall inflammation by suppressing macrophage NF-κB activation and reducing oxidative stress. Further, we explored a probable mechanism and found that the protective effect of AS-IV in AAA may correlate with upregulated AKT phosphorylation. Although the role of AKT in inflammation remains controversial, in this study we demonstrated that AKT could be anti-inflammatory in activated macrophages associated with AAA.

## Ethics Statement

The study conformed to the China’s Animal Protection Law. The Animal Care and Use Committee at the Wenzhou Medical College approved the study. Animal care complied with the Guide for the Care and Use of Laboratory Animals No. wzdw2015-0007.

## Author Contributions

JNW drafted the manuscript and prepared the figures. JNW, YZ, and SW performed the experiments and analyzed the data. KH, ST, LT, JW, YS, and JSW provided suggestions and reviewed the manuscript. YX supervised the study. KJ designed the study, obtained the funding, and supervised the whole project.

## Conflict of Interest Statement

The authors declare that the research was conducted in the absence of any commercial or financial relationships that could be construed as a potential conflict of interest.
